# Intranuclear Crosstalk between Extracellular Regulated Kinase1/2 and Signal Transducer and Activator of Transcription 3 Regulates JEG-3 Choriocarcinoma Cell Invasion and Proliferation

**DOI:** 10.1155/2013/259845

**Published:** 2013-10-30

**Authors:** Diana M. Morales-Prieto, Stephanie Ospina-Prieto, Wittaya Chaiwangyen, Maja Weber, Sebastian Hölters, Ekkehard Schleussner, Justine S. Fitzgerald, Udo R. Markert

**Affiliations:** Placenta Lab, Department of Obstetrics, University Hospital Jena, Bachstraße 18, 07743 Jena, Germany

## Abstract

Invasiveness of trophoblast and choriocarcinoma cells is in part mediated via leukemia inhibitory factor- (LIF-) induced activation of signal transducer and activator of transcription 3 (STAT3). The regulation of STAT3 phosphorylation at its ser727 binding site, possible crosstalk with intracellular MAPK signaling, and their functional implications are the object of the present investigation. JEG-3 choriocarcinoma cells were cultured in presence/absence of LIF and the specific ERK1/2 inhibitor (U0126). Phosphorylation of signaling molecules (p-STAT3 (ser727 and tyr705) and p-ERK1/2 (thr 202/tyr 204)) was assessed per Western blot. Immunocytochemistry confirmed results, but also pinpointed the location of phosphorylated signaling molecules. STAT3 DNA-binding capacity was studied with a colorimetric ELISA-based assay. Cell viability and invasion capability were assessed by MTS and Matrigel assays. Our results demonstrate that LIF-induced phosphorylation of STAT3 (tyr705 and ser727) is significantly increased after blocking ERK1/2. STAT3 DNA-binding capacity and cell invasiveness are enhanced after LIF stimulation and ERK1/2 blockage. In contrast, proliferation is enhanced by LIF but reduced after ERK1/2 inhibition. The findings herein show that blocking ERK1/2 increases LIF-induced STAT3 phosphorylation and STAT3 DNA-binding capacity by an intranuclear crosstalk, which leads to enhanced invasiveness and reduced proliferation.

## 1. Introduction

Embryo implantation is a decisive stage in the establishment of human and murine pregnancy and is accomplished when trophoblast cells invade into uterine tissue [1, 2]. An intricate interplay of cytokines, growth factors, and hormones secreted into the fetomaternal interface tightly controls this process [3]. Leukemia inhibitory factor (LIF), a member of the interleukin-6 (IL-6) family, is a cytokine which seems to play a pivotal role in human and murine reproduction [2–5]. Although LIF is mainly recognized for its regulatory functions of inflammatory cell responses in several cell types [6, 7], it also controls uterine receptivity for blastocyst implantation and influences trophoblast behavior by promoting proliferation, invasion, and differentiation in mice and humans [8, 9]. 

LIF triggers its effects by induction of a signaling heterodimer receptor consisting of the specific LIF receptor and its subunit GP130 [2]. This activates the RAS/mitogen activated protein kinase (RAS/MAPK) and janus kinase/signal transducer and activator of transcription (JAK/STAT) cascades [10–12]. In short, STATs are a family of cytoplasmic transcription factors which form hetero- or homodimers upon activation and translocate into the nucleus to influence target gene expression, such as suppressor of cytokine Signaling 3 (SOCS3), a negative feedback molecule [13, 14]. STATs are associated with regulation of implantation, placentation, and maternal immune response in early pregnancy in humans and mice [15–17]. We have demonstrated in the past that STAT3, a member of the STAT family, plays a crucial role in regulating LIF-mediated trophoblast invasion [9, 18, 19]. 

On the other hand, MAPKs are a group of protein kinases that play an essential role in signal transduction pathways modulating gene transcription in the nucleus in response to changes in the cellular environment [20]. Numerous mitogens, growth factors, and cytokines trigger their effects through ERK1/2, thus contributing not only to normal cell growth, but also to malignant transformation [21]. A recent study has demonstrated that LIF induces proliferation in the extravillous trophoblastic cell line, HTR8/svneo, via phosphorylation of ERK1/2 [22]. Similarly, decreased Akt and ERK1/2 are associated with developmental restriction of dexamethasone-induced rat placenta [23]. Altogether, these studies highlight the importance of ERK1/2 in pregnancy.

Crosstalks between the JAK/STAT and MAPK pathways have been described as occurring regularly: SOCS3 binds and inactivates RasGAP, a negative regulator of Ras signaling, leading to increased Ras/MAPK pathway activity [13]. Conversely, in other cell systems, such as in thyroid carcinoma, activated MAPKs enhance transcriptional activity of STATs by specifically phosphorylating a serine residue near its C-terminus [24]. Full activation of STAT3 requires phosphorylation at its tyr705 and ser727 residues, which allows it to dimerize and translocate into the nucleus [25, 26]. Ser727 phosphorylation is stimulus-regulated and its presence is necessary for complete STAT3 activation during oncogenesis [27]. Additionally, its inhibition decreases DNA-binding activity of STAT3 after stimulation with IL-6 [14, 28]. To date, conflicting evidence exists concerning the kinase responsible for STAT3 (ser727) phosphorylation. Some members of the MAPK family, such as Protein kinase C, Jun N-terminal kinase, extracellular signal-regulated kinase1/2 (ERK1/2), p38, and mammalian target of rapamycin (mTOR), seem to be involved, but their implications remain unclear [25, 26, 29, 30]. The apparent divergence of results may be due to the variation of cell systems and stimuli employed in the different studies. 

Taken together, a better understanding of functional trophoblast regulation seems to require further investigation of the intracellular mechanisms which govern STAT3. 

This study was performed to assess the phosphorylation of ERK1/2 and STAT3, especially with regard to serine727 phosphorylation in JEG-3 choriocarcinoma cells after stimulation with LIF and the possible crosstalk between these molecules at cytoplasmic and nuclear levels. We further aimed to detect influences that these pathways have on JEG-3 invasion and proliferation by inhibiting ERK1/2 with U0126, a specific blocker of mitogen-activated protein kinase kinase (MEK) that phosphorylates ERK1/2.

## 2. Materials and Methods

### 2.1. Cell Culture

JEG-3 choriocarcinoma cells (DSMZ, Braunschweig, Germany) were cultured in Dulbecco's modified Eagle's medium-F12 (GIBCO), supplemented with 10% heat-inactivated fetal bovine serum (FBS; SIGMA, St. Louis, USA) and 1x Penicillin/Streptomycin (PAA Laboratories; Pasching, Austria), and maintained under standard conditions (37°C, 5% CO_2_, humidified atmosphere). 

### 2.2. Protein Isolation

For protein analysis, cells were seeded in 6-well plates to reach 60–70% confluence. The succeeding morning, cells were starved for 2 h in serum-free medium and, subsequently, incubated with or without 10 mM of the chemical MEK inhibitor U0126 (Cell Signaling, Boston, USA) for another 2 h. Following this treatment, cells were challenged with 10 ng/mL LIF (Millipore, Schwalbach, Germany), washed in PBS, harvested, and lysed in cell lysis buffer supplemented with protease inhibitors (Cell Signaling). Three freeze-thaw cycles in liquid nitrogen were performed to ensure the complete lysis of cells. After centrifugation (18,000 rpm, 30 min, 4°C), supernatants were collected and protein concentrations were determined by using a Bradford-based Bio-Rad Protein Assay (BIO-RAD, Munich, Germany).

### 2.3. SDS-PAGE and Western Blotting

20 *μ*g of protein lysates was suspended in gel-loading buffer (62.5 mM Tris-HCl; pH 6.8; 2% SDS; 25% glycerol; 1% phenol blue; 5% *β*-mercaptoethanol), boiled for 7 min, and resolved on 7.5% acrylamide SDS gels. Proteins were then transferred to a hydrophobic polyvinylidene difluoride membrane (Hybond-P; GE Healthcare, Freiburg, Germany). After protein transfer, membranes were blocked in milk-containing buffer for 1 h (1x TBS containing 0.1% Tween-20 with 5% w/v nonfat dry milk). Antibodies against p-STAT3 (ser727), p-STAT3 (tyr705), p-ERK1/2 (thr 202/tyr 204), STAT3, ERK1/2, and *β*-actin (Cell Signaling) were applied in a 1 : 1000-dilution over night at 4°C. Membranes were then washed (1x TBS containing 0.1% Tween-20) and incubated with peroxidase-conjugated anti-rabbit IgG antibody (Cell Signaling) used in a 1 : 10000 dilution for 1 h at room temperature. For detection, a luminol-based system (LumiGlo; Cell Signaling) was used as described in the instructions of the manufacturer.

### 2.4. Immunocytochemistry

Cells were trypsinized, centrifuged, and resuspended in 500 *μ*L medium. Slides were washed and sterilized with ethanol, coated with cells and incubated over night at 37°C. Subsequently, fresh medium supplemented with or without 10 mM U0126 was applied for 2 h, followed by stimulation with 10 ng/mL LIF. Staining of cells was performed by using a Vectastain Elite ABC Kit (Vector Laboratories, Burlingame, USA) as follows: cells were fixed in ethanol/methanol 1 : 1 for 5 min, washed in 0.1 M PBS, and nonspecific antigens were blocked with normal goat serum for 20 min at room temperature. After blocking, slides were incubated 1 h with the primary antibody diluted 1 : 100 (p-ERK) or 1 : 200 (p-STAT3 (tyr705 or ser727)) in Antibody Diluent (DAKO, Hamburg, Germany), washed again, and incubated 30 min with biotinylated affinity-purified anti-rabbit-IgG (Cell Signaling). Thereafter, slides were treated with a solution of Avidin/Biotinylated enzyme Complex (ABC; Thermo Fisher Scientific, Bonn, Germany) for 30 min, followed by 2 min staining with 3,3 diaminobenzidine (DAB; Dako), and cell nuclei were stained with hematoxylin for 2 min. Finally, slides were dehydrated by an ethanol-to-xylene treatment, covered with Histofluid (Paul Marienfeld, Lauda-Königshofen, Germany), and stored at 4°C. Analysis was performed at a microscope Axioplan 2 (Carl Zeiss, Jena, Germany).

### 2.5. DNA Binding Capability Assay

JEG-3 cells were grown to subconfluence, serum-starved for 2 h, and then treated or not with 10 nM U0126 and 10 ng/mL LIF as for the previously described experiments. From these cells, nuclear extracts were prepared by using the Nuclear Extract Kit (Active Motif, Carlsbad, USA). Briefly, cells were collected in ice-cold PBS in the presence of phosphatase inhibitors, resuspended in hypotonic buffer, and treated with detergent to separate the cytoplasmic fraction from nuclei by centrifugation. The nuclei were then lysed and nuclear proteins were solubilized in lysis buffer. 

STAT3 DNA-binding capability was measured by using the TransAM STAT3 Kit (Active Motif). In brief, 10 ng nuclear extracts were incubated with immobilized oligonucleotides specific for STATs. STAT3 bound to DNA was then detected through use of an anti-STAT3 antibody and a secondary antibody conjugated to horseradish peroxidase (HRP), followed by a colorimetric reaction. STAT3-DNA binding was spectrophotometrically quantified in a SPECTROstar Omega (BMG Labtech, Offenburg, Germany) [31]. 

### 2.6. Cell Viability Assay

The effect of LIF and U0126 on JEG-3 cell viability was analyzed by using a Cell Titer AQeous MTS assay (Promega, Mannheim, Germany) according to the manufacturer's instructions. Assays were commenced with 1 × 10^4^ cells/well in 96-well plates. Cells were cultured in serum containing F12 medium in presence or absence of 10 ng/mL LIF and 10 mM U0126. Cell proliferation was measured in triplicates after 0, 24, and 48 h incubation by adding 20 *μ*L/well methyl tetrazolium salt (MTS) solution and measuring the absorbance at 490 nm on the previously mentioned spectrometer. 

### 2.7. Cell Invasion Assay

Cell invasion assays were conducted by using BD Matrigel Growth Factor Reduced Matrix (BD Biosciences, Heidelberg, Germany) according to the manufacturer's instructions. Hanging Cell Culture Inserts (Millicell; Millipore) were coated with Matrigel matrix (1 : 3 dilution in F12 serum-free medium) and incubated 30 min at 37°C to form a semisolid gel matrix. 5 × 10^4^ JEG-3 cells were suspended in 500 *μ*L of serum-free medium (containing or not LIF and U0126) and seeded into the upper chamber of inserts on the gel matrix. 500 *μ*L of the, respectively, identical medium was also filled into the bottom of the well. The chambers were incubated 24 h at 37°C. After incubation, cells on the upper side of the filter were removed by using cotton swabs. Cells that had invaded to the underside of the filter were first fixed with precooled 80% ethanol (20 min at 4°C), then stained with 0.1% crystal violet (5 min), and rinsed with water. The dried inserts were destained with acetic acid 10% and the absorbance was measured at 630 nm. 

### 2.8. Small Interfering RNA Treatment

Alternatively to the MEK inhibitor, JEG-3 cells were treated with predesigned small interfering RNA (siRNA) for ERK1/2 (Ambion). The following is the 5′-3′ oligonucleotide sequences: Sense: GCAGCUGAGCAAUGACCAUtt and Antisense: AUGGUCAUUGCUCAGCUGCtg. STAT-3 DNA binding capacity was measured after 24 hours of transfection. Briefly, cells were seeded in 6-well plates to reach 40–60% confluence. The next morning cells were washed with OPTIMEM (GIBCO) and 800 *μ*L fresh OPTIMEM was added. Transfections were performed with Oligofectamine (Invitrogen) as suggested by the manufacturer. Concentrations of oligonucleotides and Oligofectamine dilution were 66 nM and 1 : 2.75, respectively. After 4 hours of treatment, transfections were stopped by addition of F-12 medium (GIBCO) containing 30% fetal bovine serum without antibiotics. 

### 2.9. Statistical Analyses

All Western blots and immunocytochemical analyses have been repeated 3 times with qualitatively similar results. For kinetics of phosphorylation intensity of ERK and STAT proteins as well as for analyses of dose dependency of LIF and U0126 on STAT3-DNA-binding, a two-tailed Pearson test was performed and the correlation coefficient (*r*) was calculated. *P* < 0.05 indicates a significant correlation between stimulation time and band intensity or positive dose-dependency, respectively. For comparison between band intensities of a concrete time point and the control, a Student's *t*-test has been done. For the other assays, statistical evaluation was performed by a Student's *t*-test (for invasion assays: *n* = 7; proliferation assays: *n* = 5) and using the software packages SPSS version 17.0 (WPSS Ltd., Surrey, UK). Differences were considered significant when *P* < 0.05.

## 3. Results

### 3.1. LIF Activates JAK/STAT and RAS/MAPK Pathways

**Figure 1:**
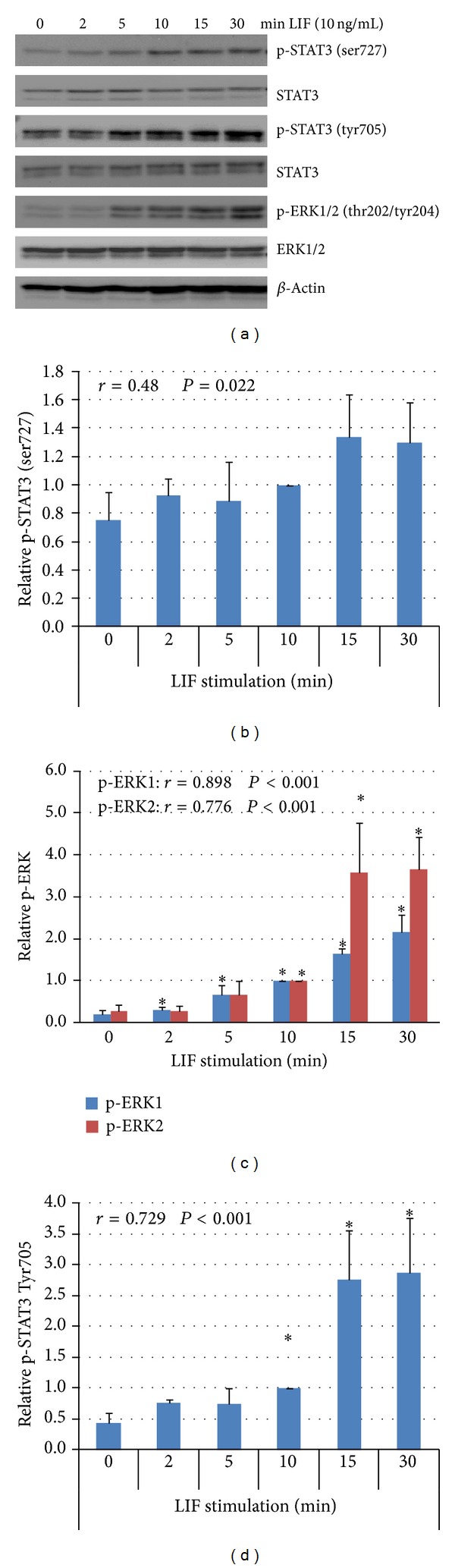
Kinetics of LIF-induced phosphorylation of STAT3 and ERK1/2 in JEG-3 choriocarcinoma cells. (a) Representative Western blot of lysates from cells starved in serum-free medium and subsequently stimulated with 10 ng/mL LIF. The bands of all blots (*n* = 3) have been scanned for density analysis. The density of bands from phosphorylated proteins ((b): phospho-STAT3 (ser727); (c): phospho-ERK1/2; (d): phospho-STAT3 (tyr705)) has been normalized against the nonphosphorylated form. The so obtained relative density at 10 min LIF stimulation has been defined as “1” and the other values have been calculated, respectively. Bars show means, error bars show standard error. *Indicates *P* < 0.05 when compared with value at 0 min LIF stimulation (Student's *t*-test). Results of an analysis of correlation after Pearson of the respective kinetics is displayed in the left upper corner of each figure. *P* < 0.05 indicates a significant correlation between stimulation time and band intensity.

Western blots demonstrated that stimulation of JEG-3 with 10 ng/mL LIF induces rapid phosphorylation (visible after 2 min) of both STAT3 phosphorylation sites (ser727 and tyr705) and ERK1/2 (thr202/tyr204). Phosphorylation remains increased during the entire analyzed period of 30 min. The positive correlation between the stimulation time and band intensity is significant for all analyzed factors (Pearson correlation). A slight constitutive phosphorylation of all factors is detectable before cells were stimulated (Figure 1).

### 3.2. LIF-Induced p-STAT3 (ser727) and Its Translocation Capacity Is ERK1/2 Independent

**Figure 2:**
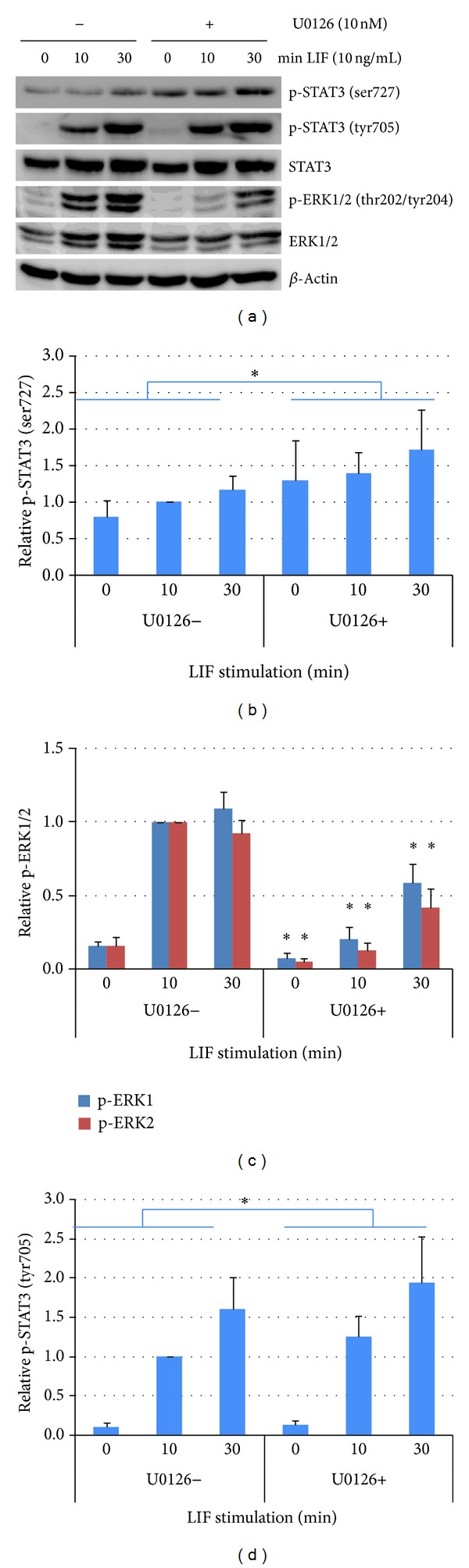
Effects of ERK1/2 blocking on LIF-induced phosphorylation of STAT3 in JEG-3 choriocarcinoma cells. (a) Representative Western blot of lysates from cells starved in serum-free medium, treated or not 2 h with the ERK1/2 blocker U0126 (10 mM), and subsequently stimulated with 10 ng/mL LIF for different time periods. The bands of all blots (*n* = 3) have been scanned for density analysis. The density of bands from phosphorylated proteins ((b): phospho-STAT3 (ser727); (c): phospho-ERK1/2; (d): phospho-STAT3 (tyr705)) has been normalized against the non-phosphorylated form. The so obtained relative density at 10 min LIF stimulation has been defined as “1” and the other values have been calculated, respectively. Bars show means, error bars show standard error. In (b) and (d) *indicates *P* < 0.05 when all band densities of U0126 treated cells were compared with all without such treatment independent from LIF stimulation time (Student's *t* test). In (c) *indicates a significant decrease when compared with the respective value without U0126 treatment.

JEG-3 cells were pretreated for 2 h with or without 10 nM U0126 and then stimulated with 10 ng/mL LIF for 10 and 30 min. As assessed by Western blotting, application of the MEK inhibitor U0126 almost completely blocks constitutive and LIF-induced ERK1/2 phosphorylation. The inhibition of MEK led to a slight but significant increase of the phosphorylation of STAT3 (ser727) and STAT3 (tyr705), when band density values from all experiments with U0126 application were compared with all experiments without U0126 independently of the LIF stimulations time (Figure 2). 

To further confirm these observations with an additional method, the phosphorylation of STAT3 and ERK1/2 in JEG-3 cells has been analyzed by immunocytochemistry before and after LIF stimulation and after the respective pretreatment with U0126. This method also allows for localization of phosphorylated factors within the cells.

**Figure 3:**
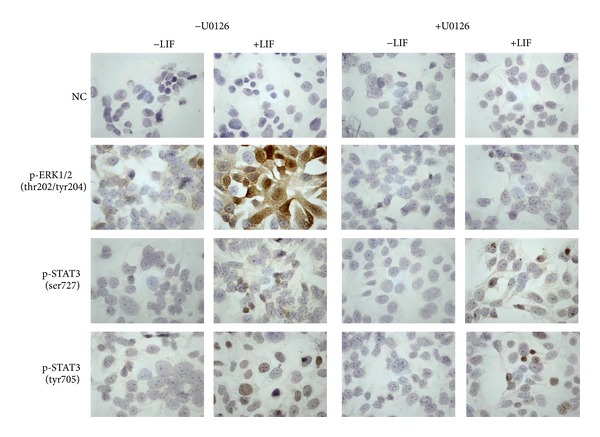
Immunocytochemistry of JEG-3 cells after incubation with U0126 and LIF. Cells were settled and incubated overnight on microscope slides, where they are attached and then treated 2 h with or without 10 mM U0126, and subsequently stimulated or not with 10 ng/mL LIF. After 15 min, cells were fixed and stained (brown) for p-ERK, p-STAT3 (tyr705), and p-STAT3 (ser727). NC: negative control.

In control cells, p-ERK1/2 is slightly detectable in the cytoplasm as well as the nucleus. After stimulation with LIF, ERK1/2 activation increases dramatically and is located mostly within the nuclei. In cells pre-treated with U0126, p-ERK is slightly visible in the nuclei of a few cells but no further activation occurs after LIF stimulation. In analogy to the Western blot observations, low levels of p-STAT3 (tyr705 and ser727) are detectable and located in the cytoplasm in control cells. Stimulation with LIF induces an increase of phosphorylation and translocation of p-STAT3 (tyr705 and ser727) into the nucleus. The slight increase of STAT3 (tyr705 and ser727) phosphorylation observed in Western blots after pre-treatment with U0126 is hardly visible with this method. The translocation of p-STAT3 into the nucleus seems to be unaffected (Figure 3).

### 3.3. ERK1/2 Inhibition Enhances Intranuclear STAT3 DNA-Binding Capability

**Figure 4:**
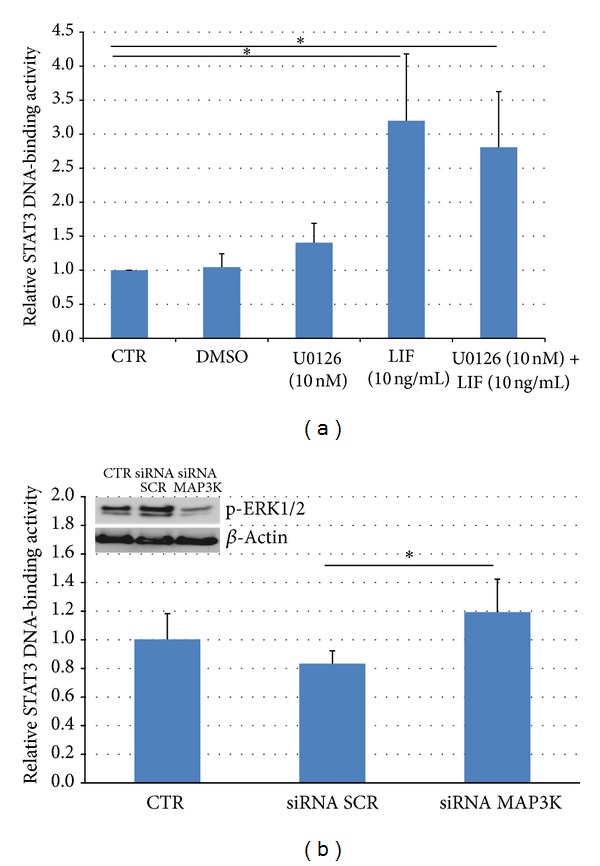
STAT3 DNA-binding capacity in JEG-3 cells after ERK1/2 inhibition and stimulation with LIF. STAT3-DNA-binding capacities were assessed by an ELISA-based colorimetric assay. (a) Starved JEG-3 cells were treated or not with U0126 (10 nM) for 2 h and then stimulated or not with LIF (10 ng/mL) for another 30 min *n* = 5. (b) ERK1/2 expression was silenced by siRNA (silencing efficiency displayed by a representative Western blot, left upper corner) *n* = 3. Bars represent the mean of independent assays. Error bars show standard error of the mean. *P* < 0.05 (Student's *t*-test).

Treatment of JEG-3 cells with U0126 alone or siRNA for ERK1/2 slightly enhances intranuclear STAT3 DNA-binding activity demonstrating a LIF-independent mechanism (Figures 4(a) and 4(b)). ERK1/2 knockdown efficiency was above 60% as assessed by Western blotting (Figure 4(b)).

The effect of U0126 or ERK1/2 siRNA transfection on STAT3 DNA-binding activity is further enhanced after additional treatment with LIF (*P* < 0.05). Nevertheless, stimulation with LIF alone results also in a significant increase of STAT3 transcriptional activity (*P* < 0.05), which is slightly higher than treatment with both LIF and U0126 (Figure 4) or LIF and siRNA (data not shown). These results demonstrate that the STAT3 transcriptional activity can be augmented upon inhibition of ERK1/2 without any additional external stimulation, but also after stimulation with LIF. Similarities between levels after treatment with either the combination of LIF and U0126 or LIF alone may be due to the bioavailability of STAT3 in the nuclei. 

### 3.4. ERK1/2 Activation Is a Major Regulator of JEG-3 Cell Viability

**Figure 5:**
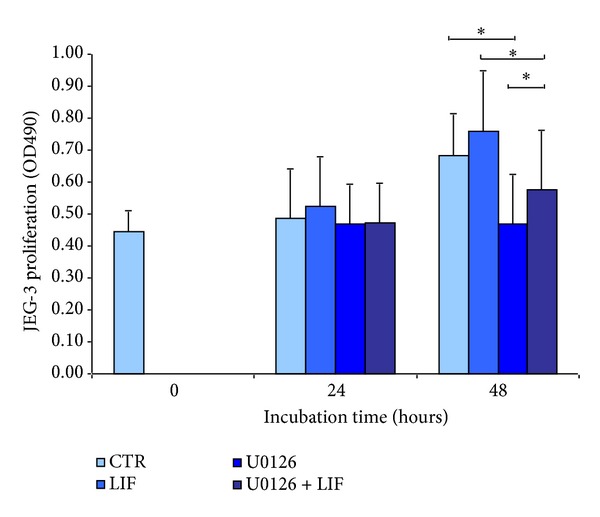
Effect of LIF and U0126 on the proliferation of JEG-3 cells. JEG-3 cells were incubated for up to 48 h in presence or absence of 10 mM U0126 and 10 ng/mL LIF. A MTS colorimetric assay was performed and optical density (OD) at 490 nm was measured to assess cell proliferation. Bars show mean values of *n* = 5 independent experiments, which have been performed in triplicates (controls in 12 replicates). Error bars indicate standard error of the mean. *Horizontal bars indicate *P* < 0.05 for comparison of the respective means, Student's *t*-test.

JEG-3 cells were cultured in presence or absence of LIF and U0126 to assess proliferation rates. For this approach, the metabolic activity was measured after 24 h and 48 h in a MTS assay. Proliferation of JEG-3 cells is obvious after 48 h and LIF slightly increases this proliferation. The application of U0126 completely inhibits the proliferation, which is significant when compared with the respective control cells after 48 h of culture. This reduction is slightly, but significantly, recovered by simultaneous treatment with LIF (Figure 5). Also in JEG-3 cells stimulated with LIF, ERK1/2 inhibition by application of U0126 leads to a significant reduction of proliferation. To exclude the possible effect of the U0126 vehicle DMSO, an independent assay was performed by adding the respective concentration of DMSO to the control cells and demonstrated that DMSO had no influence on proliferation (data not shown). 

### 3.5. Blocking ERK1/2 Increases JEG-3 Cell Invasion

**Figure 6:**
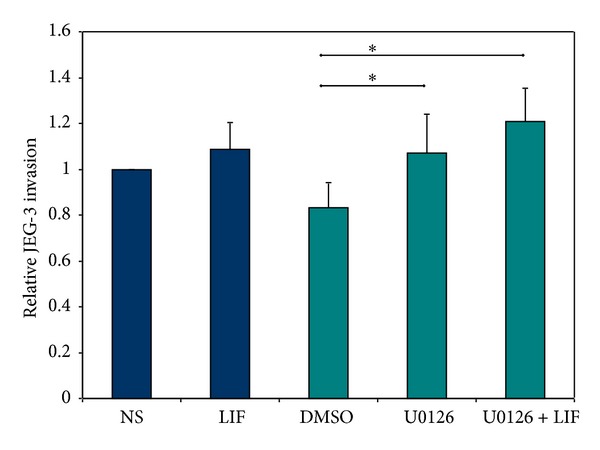
Relative invasiveness of JEG-3 cells upon ERK1/2 inhibition and stimulation with LIF. JEG-3 cells were seeded on Matrigel-coated transwell chambers in presence or absence of 10 nM U0126 and 10 ng/mL LIF as indicated. Relative invasion was assessed after 24 h as described in materials and methods and measured as absorbance at 630 nm. Results were normalized to nonstimulated cells and are expressed as mean ± standard error (*n* = 7). *P < 0.05; Student's *t* test (two-tailed).

LIF induces approximately 15% increase in invasiveness of JEG-3 cells through Matrigel, similar to previously published data. DMSO decreases the invasiveness of control cells slightly by 9%. Administration of U0126 (dissolved in DMSO) results in a significant 32% enhancement of the invasive activity. The combined application of LIF and U0126 also induces a significant increase of invasiveness compared with the respective control cells, which is slightly higher than the application of both factors separately (Figure 6). These results correlate with the previously described increased STAT3 (tyr705 and ser727) phosphorylation and the STAT3-DNA-binding capacity after blocking ERK1/2.

## 4. Discussion

Activation of MAP kinases and JAK/STAT cascades is related to carcinogenesis and proliferation in numerous cell types including trophoblast cells and their malign derivatives [1, 32]. Previously, we demonstrated that LIF exerts a dose-dependent effect on STAT3 (tyr705) activation, more intensively than other members of the IL-6 family of cytokines [19]. It is also known that IL-6-like cytokines activate the MAPK pathway in several cell types [33, 34]. Here, we demonstrate that LIF triggers phosphorylation of both pathways simultaneously within 5 min of stimulation, which indicates that activation of both is independent of each other. 

Complete STAT3 activation is dependent on the phosphorylation at the ser727 and tyr705 amino acid residues [14, 25]. Since STAT3 contains a characteristic ERK-MAPK phosphorylation site (-pro-X-ser/thr-pro-), ERK was expected to phosphorylate the ser727 residue of STAT3 [35]. Therefore, we focused on the effect of ERK1/2 inhibition on STAT3 (ser727) phosphorylation. We have chosen to perform the current study on the JEG-3 choriocarcinoma cell line and not on the immortalized first trimester trophoblast cell line HTR8/SVneo because recently several reports remark major differences between HTR8/SVneo cells, primary trophoblast cells, and choriocarcinoma cell lines [36, 37].

In JEG-3 cells, ERK1/2 phosphorylation was not necessary for phosphorylation of either STAT3 (ser727) or STAT3 (tyr705) induced by LIF as demonstrated by Western blotting and immunocytochemistry. These results correspond with a report on HepG2 hepatocellular carcinoma cells in which IL-6-induced STAT3 (ser727) phosphorylation was also ERK1/2 independent [25]. The variety of information from the literature indicates that the kinase responsible for STAT3 (ser727) phosphorylation depends on the individual cellular context and the respective stimulus. Several protein kinases such as protein kinase C, Jun N-terminal kinase, p38, and mTOR may also be responsible for STAT3 (ser727) phosphorylation [24, 26, 30, 38–40]. Experimental evidence for this association was previously reported since mTOR is required for the constitutive phosphorylation of STAT3 (ser727) in the immortalized first-trimester trophoblast cell line HTR8/SVneo [30]. 

In accordance with our previous investigations based on electrophoretic mobility shift assays [19], we now observed an increase in the DNA-binding capacity of STAT3 after stimulation with LIF by using an alternative method (TransAM STAT3 kit; see Section 2). In the current study, the LIF-independent DNA-binding activity of STAT3 increased when activation of ERK1/2 was abrogated either by treatment with the chemical inhibitor U0126 or by siRNA knockdown. These results demonstrate that activated ERK1/2 functions as an inhibitor of the transcriptional activity of STAT3. This coincides with a report in LU1205 melanoma cells, in which STAT3 transcriptional activities can be activated upon inhibition of ERK and constitutively active ERK signaling resulted in downregulation of STAT3 and STAT5 transcriptional activities [41].

To decipher functional correlates with the biochemical findings, we analyzed the effects of ERK1/2 blocking on proliferation and invasion of JEG-3 cells. STAT3 activation induced by LIF enhances trophoblastic cell proliferation and invasion [19, 22]. This elevated JEG-3 proliferation in the presence of LIF is employed as base for our current investigation. Blocking ERK1/2 significantly reduces proliferation, similar to the results recently published on HTR8/SVneo cells [22], while raises STAT3 DNA-binding ability as mentioned previously. This indicates that ERK rather than STAT3 is responsible for proliferative effects in JEG-3. Also in line with our findings, we have previously reported that other pathways, which alter STAT3 (ser727) phosphorylation, are also involved in the regulation of proliferation in trophoblastic cells (HTR8/SVneo cells) [30].

We have previously reported that several members of the IL-6 family of cytokines induce invasion of trophoblastic cells and that IL-6 receptor-mediated STAT3 activation and translocation into the nucleus are essential for mediating the invasion promoting effects of LIF, IL-11, and IL-6 in trophoblast and choriocarcinoma cells [18, 42, 43]. In the current study, we have shown that inhibition of ERK induces an increase in the transcriptional activity of STAT3 and this crosstalk results in enhanced cell invasion. 

## 5. Conclusions

**Figure 7:**
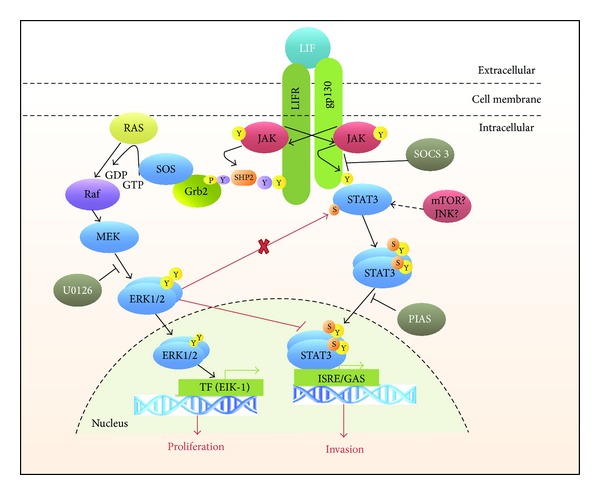
Scheme of the proposed LIF signaling pathway in trophoblasts. Red lines demonstrate the major findings of this paper: LIF triggers activation of JAK/STAT and MAPK pathways independently. ERK1/2 does not induce STAT3 (ser727) phosphorylation but antagonizes STAT3 DNA-binding capacity in the nucleus. JAK/STAT and MAPK activation result in different cell-responses: increase of invasiveness and proliferation, respectively.

Summarized, the here in presented findings demonstrate that in JEG-3 choriocarcinoma cells, LIF simultaneously employs two main intracellular signaling cascades, the JAK/STAT and MAPK pathways. ERK1/2 does not induce STAT3 phosphorylation but, instead, represses STAT3 (tyr705 and ser727) phosphorylation and antagonizes STAT3 DNA-binding capacities in the nucleus (Figure 7). 

Both pathways seem to have different functions. ERK1/2 is a major, but not sole, promoter of JEG-3 proliferation and is a negative regulator of STAT3, while STAT3 rather induces invasion (Figure 7). It may be concluded that dysfunctions of both pathways may be involved in placentation disorders and trophoblast malignancies. The better understanding of the role of individual factors may lead to the development of new therapeutic strategies.
